# Comparison of the fixation ability between lag screw and bone plate for oblique metacarpal shaft fracture

**DOI:** 10.1186/s13018-022-02963-3

**Published:** 2022-02-05

**Authors:** Yung-Cheng Chiu, Tsung-Yu Ho, Cheng-En Hsu, Yen-Nien Ting, Ming-Tzu Tsai, Jui-Ting Hsu

**Affiliations:** 1 School of Medicine, China Medical University, Taichung, 404 Taiwan, ROC; 2 Department of Orthopedic Surgery, China Medical University Hospital, Taichung, 404 Taiwan, ROC; 3 Department of Orthopaedics, Taichung Veterans General Hospital, Taichung, 407 Taiwan, ROC; 4 Sports Recreation and Health Management Continuing Studies-Bachelor’s Degree Completion Program, Tunghai University, Taichung, 407 Taiwan, ROC; 5 3D Printing Medical Research Center, China Medical University Hospital, Taichung, 404 Taiwan, ROC; 6 Department of Biomedical Engineering, Hungkuang University, Taichung, 433 Taiwan, ROC; 7 School of Dentistry, College of Dentistry, China Medical University, 91 Hsueh-Shih Road, Taichung, 40402 Taiwan, ROC; 8 Department of Bioinformatics and Medical Engineering, Asia University, Taichung, 413 Taiwan, ROC

**Keywords:** Oblique metacarpal shaft fracture, Screw, Regular plate, Locked plate

## Abstract

**Background:**

For oblique metacarpal shaft fracture, if anatomical reduction is achieved through conservative cast immobilization rather than stable fixation, bone malrotation can easily occur, resulting in severe loss in hand prehensile function. However, whether bone plate fixation or only lag screw fixation is more preferable remains unclear. Few studies have evaluated whether screw fixation can provide biomechanical fixation strength similar to bone plate fixation.

**Objective:**

We assessed the difference in fixation strength between fixtation with two lag screws and bone plate for oblique metacarpal shaft fractures.

**Materials and methods:**

We created oblique metacarpal shaft fractures on 21 artificial bones and fixated them using (1) double lag screw (2LS group), (2) regular plate (RP group), or (3) locked plate (LP group). To obtain the force–displacement data, a cantilever bending test was conducted for each specimen through a material testing machine. One-way analysis of variance and a Tukey test were conducted to compare the maximum fracture force and stiffness of the three fixation methods.

**Results:**

The maximum fracture force of the 2LS group (mean + SD: 153.6 ± 26.5 N) was significantly lower than that of the RP (211.6 ± 18.5 N) and LP (227.5 ± 10.0 N) groups (*p* < 0.001). However, no significant differences were discovered between the RP and LP groups. The coefficient of variation for the maximum fracture force of the 2LS group (17.3%) was more than twice as high as that of the RP (8.7%) and LP (4.4%) groups. In addition, the stiffness of the three fixation methods was similar.

**Conclusion:**

Compared with bone plate fixation, double lag screw fixation yielded slightly lower maximum bearable fracture force but similar stiffness. Therefore, this technique could be used for treating oblique metacarpal shaft fractures. However, using double lag screw fixation alone is technically demanding and requires considerable surgical experiences to produce consistent results.

## Introduction

Metacarpal fractures are common, accounting for approximately 36%–42% of hand trauma cases [1]. Metacarpal neck fracture is the most common type of metacarpal fracture, which is twice as common as metacarpal shaft fracture [2]. However, unlike metacarpal neck, which is mostly composed of cancellous bone, the metacarpal shaft mainly comprises cortical bone and thus is more difficult to heal and requires surgical treatment for bone union [3]. According to different injury mechanisms, metacarpal shaft fractures result in different forms: axial loading and direct blow tend to cause transverse or comminuted fractures, whereas torsion leads to oblique or spiral fractures, among which torsion accounts for 75% of the causes of metacarpal shaft fractures [4]. Stable, nondisplaced metacarpal shaft fractures can be treated conservatively through cast immobilization. However, fractures with larger displacement and greater degrees of angulation are unstable because of the continual traction force generated by the interosseous muscle and the limited bony contact area at the fracture site. If conservative treatment is adopted in such cases, satisfactory anatomic reduction is difficult to achieve, the probability of nonunion becomes high, and bone malrotation can easily occur. This leads to scissoring deformity of the fingers during hand grasping and severe loss of hand prehensile function [5]. Therefore, oblique and spiral fractures typically require surgical fixation.

Oblique metacarpal shaft fractures are commonly fixed using bone plate or lag screws [6]. However, which of them is superior remains unclear. In general, lag screw fixation is minimally invasive, cost effective and time saving [7, 8]. Nevertheless, surgeons have concerns over it because of its mechanical stability and is a relatively challenging technique. Surgeons need to overcome the steep learning curve of lag screw fixation technique to reduce postoperative complications. By contrast, bone plate fixation provides superior fixation strength and is easier to operate. It enables earlier range of motion and earlier start for the subsequent rehabilitation program, resulting in a more satisfactory treatment result. However, it is also likely to cause complications, such as metacarpophalangeal joint stiffness, extensor tendon adhesion, and iatrogenic injury of the dorsal cutaneous branch of the ulnar or radial nerve, whether a regular or a locked plate is applied. In addition, the cost of such surgery is high.

Studies have discussed the fixation of transverse metacarpal shaft fractures [9–11], but few have explored that of oblique fractures [6]. Therefore, in this mechanicstic study, we compared the stability of double lag screw fixation and bone plate fixation to determine the optimal treatment for oblique metacarpal shaft fractures.

## Materials and methods

### Specimen preparation

**Fig. 1:**
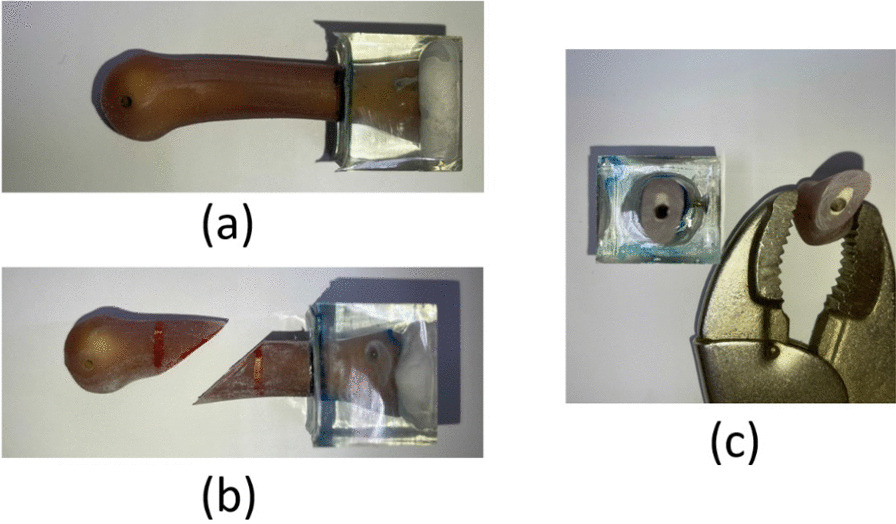
Artificial metacarpal bones (**a**) intact and with oblique metacarpal shaft fracture in **b** lateral view and **c** cross-sectional view

We selected 14 artificial third metacarpal bones (Sawbones, Vashon, WA, USA). A 0.8-mm chainsaw was used to create oblique metacarpal shaft fractures on the artificial metacarpal bones (Fig. 1). Epoxy embedding and fixation were conducted at the proximal end of the artificial metacarpal bones.

### Fixation approaches

We created oblique fractures on 21 artificial metacarpal bones in total and evenly divided them into three groups: (1) double lag screw fixation (the 2LS group), (2) regular plate fixation (the RP group), and (3) locked plate fixation (the LP group). The fracture fixation was performed by Yung-Cheng Chiu, a senior hand surgeon.

**Fig. 2:**
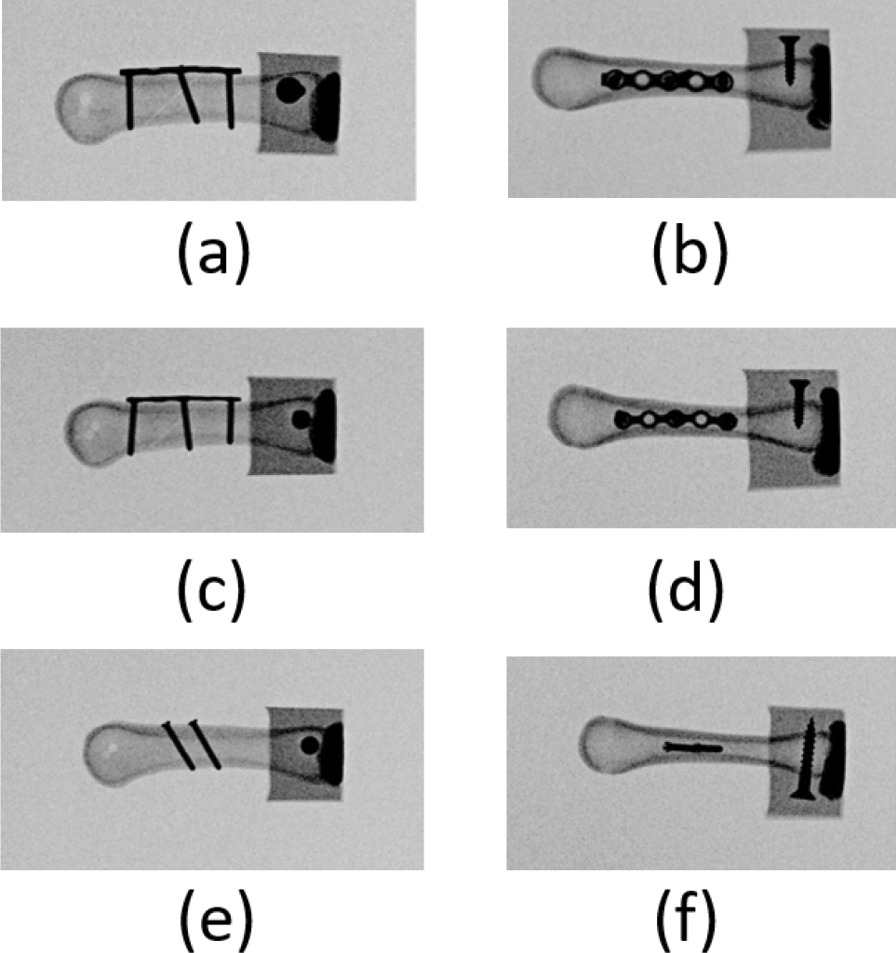
Radiographs of the three fixation approaches: regular plate fixation in **a** anterior–posterior view and **b** lateral view; locked plate fixation in **c** anterior–posterior view and **d** lateral view; and double screw fixation (**e**) anterior–posterior view and **f** lateral view

Double lag screw fixation: After anatomical reduction, seven obliquely fractured artificial bones were fixed by placing two 2.3-mm parallel cortical screws (Stryker, Germany) into the bones from the dorsal cortex and perpendicularly to the fracture line until they penetrated the far cortex to stabilize the fracture (Fig. 2).

Regular plate fixation: After anatomical reduction, seven obliquely fractured artificial bones were fixed by placing a regular 5-hole plate (Stryker, Germany) on the dorsum of the metacarpal shaft and locking a cortical screw each at the proximal point, in the middle, and at the distal point of the fracture site. The screws were inserted vertically to the anatomical axis of the metacarpal bone until they penetrated the near and far cortex (Fig. 2).

Locked plate fixation: After anatomical reduction, seven obliquely fractured bones were fixed by placing a locked 5-hole plate (Stryker, Germany) on the dorsum of the metacarpal shaft and locking a locked screw each at the proximal point, in the middle, and at the distal point of the fracture site. The screws were inserted vertically to the anatomical axis of the metacarpal bone until they penetrated the near and far cortex.

### Biomechanical test

**Fig. 3:**
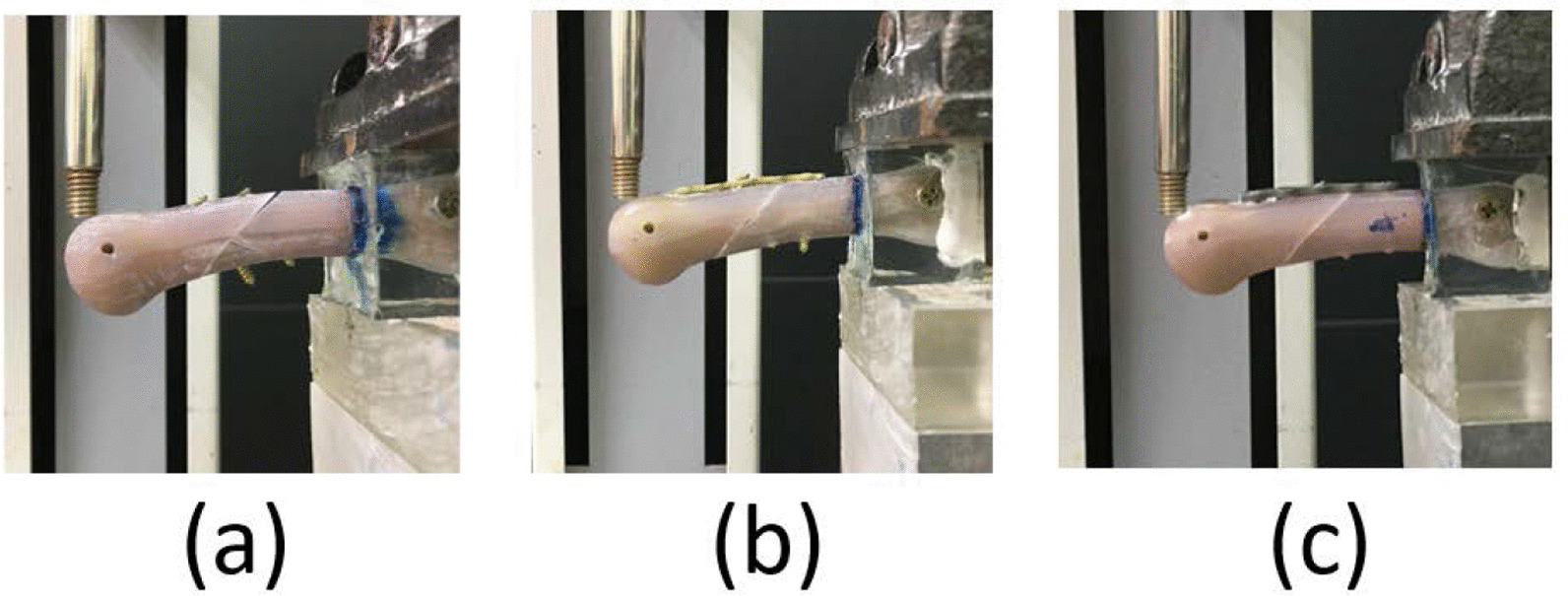
The in vitro biomechanical tests for the three fixation approaches: **a** double lag screw fixation; **b** regular plate fixation; and **c** locked plate fixation

The in in vitro test was conducted as described in our previous studies [7, 8, 12–14]. The in vitro mechanical test was conducted using cantilever bending tests. The material testing system employed was JSV-H1000 (Japan Instrumentation System, Nara, Japan) (Fig. 3). A force at a decreasing rate of 10 mm/min was applied to the distal region on the dorsal side of the artificial metacarpal bones until they fractured. The force–displacement curve generated during the force application was recorded, and the maximum fracture force and stiffness were obtained accordingly.

### Statistical analysis

The maximum fracture force and stiffness of the three fixation methods for oblique metacarpal shaft fractures are expressed as mean ± standard deviation. One-way analysis of variance with a post hoc Tukey test were used to compare the fixation ability of the three methods. SPSS 19.0 (IBM, Armonk, NY, USA) was used for statistical analysis, and a *p* value less than 0.05 was set as statistically significant.

## Results

The maximum fracture force and stiffness of the three fixation methods are presented in Table 1. The maximum fracture force of the 2LS group (153.6 ± 26.5 N) was significantly lower than that of the RP (211.6 ± 18.5 N; difference: 37.7%) and LP groups (227.5 ± 10.0 N; difference: 48.1%) (* p*< 0.05). However, no significant differences were observed between the RP and LP groups. In addition, for the maximum fracture force, the coefficient of variation of the 2LS group (17.3%) was more than twice as high as that of the RP (8.7%) and LP (4.4%) groups. Although the mean stiffness of the 2LS group (57.0 ± 8.2 N/mm) was lower than that of the RP (64.7 ± 10.2 N/mm) and LP (65.4 ± 8.2 N/mm) groups, no significant differences were observed among the three groups.

**Table 1:** Maximum fracture force and stiffness of the three fixation types for oblique metacarpal shaft fracture

Parameters (unit)	Value	Three fixation approaches			
		2LS	RP	LP	*P*^†^
Maxi fracture force (N)	Mean*	153.6^a^	211.6^b^	227.5^b^	< 0.001
	SD	26.5	18.5	10.0	
	Max	198.7	236.9	227.1	
	Min	118.2	184.8	194.6	
Stiffness (N/mm)	Mean*	57.0^a^	64.7^a^	65.4^a^	0.222
	SD	8.2	10.2	8.2	
	Max	70.1	78.1	76.1	
	Min	49.0	42.1	51.3	

*2LS* Double lag screw fixation, *RP* Regular plate fixation, *LP* Locked plate fixation, *SD*Standard deviation, *Max* Maximum, *Min* Minimum

^†^One-Way ANOVA test

*Post hoc pairwise comparisons were conducted using Tukey’s test. For each parameter of max fracture force or stiffness, the means with the same letter (a or b) are not significantly different

## Discussion

Clinically, surgical methods have been proposed for fixation of oblique metacarpal shaft fractures [6]. Bone plate fixation provides superior fixation strength, is easier to perform, and facilitates an earlier range of motion and start for rehabilitation programs, thereby resulting in a more satisfactory treatment outcome. However, postoperative complications, such as metacarpophalangeal joint stiffness, extensor tendon adhesion, and iatrogenic injury of the dorsal cutaneous branch of the ulnar or radial nerve, are likely to occur, and the cost of the surgery is relatively high. By contrast, lag screw fixation reduces these complications and is minimally invasive, cost effective, and time saving [8, 13]. Nevertheless, according to literature and clinical experience, lag screw fixation may not provide adequate biomechanical fixation strength [15–18]. In the present study, we explored the fixation strength using lag screw and bone plate fixation for oblique metacarpal shaft fractures and observed that the maximum bearable fracture force of double lag screw fixation was slightly lower than that of bone plate fixation, but the stiffness of the two was comparable. Hence, double lag screw fixation is feasible for treating oblique metacarpal shaft fractures. However, use of only the double lag screw fixation technique requires more experience to achieve consistent results.

Oblique and spiral fractures account for a high proportion of metacarpal shaft fractures. Because of the traction force generated from the interosseous muscle, the anatomical parts of oblique and spiral fractures can cause greater displacement at the fractured bone end, angulation deformity, shortening of metacarpal lengths because of the overlapping at the fractured bone end, and malrotation. In addition, such fractures are relatively unstable due to the limited bony contact area at the fracture site. Therefore, if conservative cast immobilization treatment is applied, a longer immobilization time is required. Patients may have to begin their rehabilitation program 6–8 weeks after immobilization, which may result in finger joint stiffness [11, 19, 20], Mal-union, nonunion, pressure skin necrosis, muscle ischemia from tightness [20]. The probability of nonunion, bone malrotation, and scissoring deformity of the fingers also increase. Every additional 5° of metacarpal shaft rotation leads to a 1.5 cm overlap at the fingertips [21]. One degree of metacarpal fracture rotation has been shown to produce 5° of fingertip rotation [1, 19] and severe loss of hand prehensile function, thus negatively affecting the patient’s quality of life. Accordingly, determining the optimal technique for fixation of oblique metacarpal shaft fractures is vital.

Previous studies have adopted in vitro experiments for metacarpal fracture fixation, human cadaveric bones [22], animal bones [7], or artificial bones [8, 12–14]. We employed artificial metacarpal bone because fresh human metacarpal bones were difficult to obtain and because some literature and the American Society for Testing and Materials had used artificial bones for experiments. Furthermore, we used a cantilever method to estimate the strength of the three fixation methods explored in the present study and adopted maximum fracture force and stiffness as the indices of fixation strength [8, 12–14].

Improvement in materials and techniques have provided orthopedic surgeons with many methods to fix oblique and unstable metacarpal shaft fractures, such as (1) K wire, (2) bone plate, (3) lag screw, and (4) intramedullary headless screw fixation [6]. Preceding biomechanical studies on metacarpal fractures have focused on transverse fractures [9, 10, 15]. However, the results of biomechanical experiments have revealed different findings in terms of fixation for oblique fracture. For example, the stiffness of crossed K-wire fixation was revealed to be equivalent to the combination of intraosseous wiring and oblique K-wiring; the stiffness of dorsal plating, with or without lag screws, was revealed to be higher than that of both aforementioned methods [15]. Accordingly, we compared fixation methods between lag screw fixation and bone plate fixation for oblique metacarpal shaft fractures.

In preceding in vitro experiments for oblique metacarpal shaft fractures, [23] applied cadaver metacarpals and 1.5-mm and 2.0-mm screws to fix oblique metacarpal fractures through bicortical interfragmentary fixation and the lag screw technique, respectively, and observed that the 1.5-mm bicortical interfragmentary screws provided sufficient fixation strength. The load to failure of the experiment was 72.31 N, which was much lower than that of double lag screw fixation in the present study (153.6 N). [22] compared the fixation strength of dorsal plating, lag screw fixation, and headless compression screw fixation and revealed that headless compression screw fixation offered the most satisfactory fixation. The maximum fracture force and stiffness of the double lag screw fixation were 234.1 N and 172.18 N/mm, respectively, which were much higher than our results (153.6 N and 57.0 N/mm, respectively). These inconsistencies were primarily because of the differences in the bone specimens used. The previous two studies employed cadaver metacarpals, which are more realistic, whereas we used artificial bones; each artificial bone specimen was identical, which explains the lower coefficient of variation in the present study. Furthermore, we conducted a cantilever bending test for a mechanical test rather than a three-point bending test employed in preceding studies.

In our mechanical experiment, the maximum fracture force of the 2LS group was nonsignificantly lower than that of the RP and LP groups. This implies that as long as patients avoid strong physical impact after the surgery, the fixation strength provided with double lag screw fixation can be adequate for metacarpal shaft fractures. Literature has also indicated that stiffness should be a more critical index for fixation strength assessment and a more reliable indication of construct stability than the maximum fracture force [14, 24], because during fracture healing, refracture of the metacarpal bone as a result of an extremely strong active or passive force does not and should never occur. In the present study, the RP and LP groups exhibited no significant difference in any aspect. In addition, the coefficient of variation of the 2LS group was more than twice as high as that of the RP and LP groups. Basically, in the same group, the larger the coefficient of variation, the worse the consistency of the experimental results, and because the artificial bone specimens used in this study were the same quality, the difference resulted from the material properties of the artificial bone itself might be excluded, so the large differences in experimental results are mainly due to inconsistency in the skill of fixation approach. Therefore, this suggested that double lag screw fixation was less fault tolerant. Even if the surgery is performed successfully, the stability would not be as high as that of the bone plate fixation. Therefore, more experienced surgeons are required to obtain optimal results when using double lag screw fixation.

Although bone plate fixation provides more stability, double lag screw fixation has other benefits: It is cost effective, is minimally invasive, and can reduce extensor tendon and sensory nerve adhesion. Moreover, our results indicated that it provided comparable stiffness to bone plate fixation. The fixation power it provides can sustain bony union. However, compared with patients treated with bone plate fixation, those who receive only screw fixation require more protection. The surgery may fail if patients receive external force damage that exceeds the maximum fracture force. Surgeons’ experience in performing lag screw fixation is critical, given that the coefficient of variation was significantly high even in the in vitro mechanical experiment where the variables were controlled, fracture pattern was consistent, and surgery was conducted by the same surgeon. Hence, caution must be applied when employing this surgical approach. Each screw should be inserted as perpendicularly to the fracture line as possible.

This study has some limitations. As encountered by many previous researchers [7, 14, 24, 25], fresh human metacarpal bones are difficult to obtain. Therefore, we used artificial bones for the experiment. However, the material properties of artificial bones are different from those of real human bones. Moreover, we used a cantilever bending test to assess fixation strength, but such a load pattern may not comprehensively simulate the actual strained condition in phalanx motions. Future studies should design more comprehensive experiments to obtain more detailed findings.

## Conclusion

This study employed artificial metacarpal specimens to explore the fixation strength of different surgical methods for oblique metacarpal shaft fractures. Although the maximum bearable fracture force of double lag screw fixation was slightly lower than that of bone plate fixation, the stiffness of the two methods was comparable. However, double lag screw fixation requires considerable surgical experience for consistent results.

## Data Availability

The data sets used and analyzed during the current study are available from the corresponding author upon reasonable request.

## Abbreviations

2 LS group: Double lag screw
RP group: Regular plate
LP group: Locked plate
ANOVA: Analysis of variance
